# Women’s health literacy and the complex decision‐making process to use complementary medicine products in pregnancy and lactation

**DOI:** 10.1111/hex.12910

**Published:** 2019-05-22

**Authors:** Larisa A. J. Barnes, Lesley Barclay, Kirsten McCaffery, Parisa Aslani

**Affiliations:** ^1^ School of Pharmacy The University of Sydney Sydney New South Wales Australia; ^2^ University Centre for Rural Health The University of Sydney Lismore New South Wales Australia; ^3^ School of Public Health The University of Sydney Sydney New South Wales Australia

**Keywords:** complementary therapies, dietary supplements, health literacy, herbal medicine, information seeking behaviour, lactation, patient‐provider relationship, pregnancy, qualitative research, self‐care

## Abstract

**Background:**

Little is known about women's decision‐making processes regarding using complementary medicine products (CMPs) during pregnancy or lactation.

**Objectives:**

To explore the decision‐making processes of women choosing to use CMPs in pregnancy and lactation; and to investigate how women's health literacy influences their decisions.

**Design, setting and participants:**

In‐depth interviews and focus group discussions were held with twenty‐five pregnant and/or breastfeeding women. Data were analysed using thematic analysis.

**Results:**

Key to women's decision making was the desire to establish a CMPs safety and to receive information from a trustworthy source, preferably their most trusted health‐care practitioner. Women wanted positive therapeutic relationships with health‐care practitioners and to be highly involved in the decisions they made for the health of themselves and their children. Two overarching components of the decision‐making process were identified: (a) women's information needs and (b) a preference for CMP use. Women collated and assessed information from other health‐care practitioners, other mothers and published research during their decision‐making processes. They showed a strong preference for CMP use to support their pregnancy and breastfeeding health, and that of their unborn and breastfeeding babies.

**Discussion and Conclusions:**

Complex decision‐making processes to use CMPs in pregnancy and lactation were identified. The participants showed high levels of communicative and critical health literacy skills in their decision‐making processes. These skills supported women's complex decision‐making processes.

## 1 INTRODUCTION

Complementary and alternative medicine (CAM) includes multiple CAM practices (therapies) as well as complementary medicine products (CMPs) like vitamin or mineral supplements or herbal medicines.^1^, ^2^, ^3^ The World Health Organization refers to CAM as ‘*a broad set of health care practices that are not part of that country's own tradition or conventional medicine and are not fully integrated into the dominant health‐care system’.* 4 Similarly, the use of most CMPs is not considered to be part of conventional biomedical practice,^3^ although some CMPs have been studied in clinical trials and subsequently have been co‐opted or included in biomedical practice (eg some herbal medicines and some probiotic strains).^5^, ^6^ CMPs like herbal medicines and nutritional supplements are commonly used in pregnancy and lactation by women around the world.^7^, ^8^, ^9^ The practice of herbal medicine is often based on traditional knowledge and use, as passed down by traditional medicine healers in different cultures.^10^ Some nutritional supplements (eg iron, folic acid and iodine supplements) are part of evidence‐based maternity care practice and are recommended in pregnancy and lactation by both medical practitioners^9^, ^11^, ^12^ and complementary medicine practitioners.^9^, ^13^, ^14^ High rates of herbal medicine use in pregnancy have been noted. One multinational study found that 28.9% of participants reported use of herbal medicines in pregnancy, with highest rates reported in Russia (69%), Eastern Europe (51.9%) and Australia (43.8%).^7^ Herbal medicine use in lactation is also common internationally (eg see refs^15^, ^16^, ^17^) and by Australian women.^18^, ^19^ Other studies have found high CMP use in Australia too: around 50% of Australian women have been shown to use herbal medicines in pregnancy, and around 90% to use vitamin or mineral supplements.^14^, ^20^

Previous research has established that women in high‐income economies use complementary medicines during pregnancy and breastfeeding for several reasons. These include the desire for self‐determination and choice in health‐care decisions,^21^, ^22^ including the desire for natural childbirth,^23^ to prepare for labour,^24^, ^25^ treat common conditions of pregnancy,^9^, ^18^, ^24^, ^25^, ^26^, ^27^ and promote their own and their babies’ health and well‐being.^8^, ^24^, ^25^ During breastfeeding specifically, herbal galactagogues are used to correct perceived or diagnosed breastmilk insufficiency, and other herbs are used to support post‐partum health and recovery after birth or to treat common conditions like mastitis or upper respiratory tract infections.^8^, ^9^, ^18^, ^19^, ^28^ Complementary or alternative medicine (CAM) use prior to pregnancy has been associated with use during pregnancy.^24^, ^25^, ^29^, ^30^ CMP use in pregnancy or breastfeeding is also associated with biomedical or CAM health‐care practitioner prescription or recommendation.^9^, ^12^ A positive relationship with their CAM practitioners has been linked to pregnant or breastfeeding women's use of CAM.^21^, ^28^ Women appreciate CAM practitioners’ holistic approaches to health, including consideration of mental‐emotional, physical, social and spiritual health.^31^, ^32^ They prefer CAM practitioners who facilitate and encourage self‐empowerment and autonomy in health care and demonstrate positive patient‐provider communication.^21^, ^31^, ^33^, ^34^

Self‐prescription of complementary medicine products (CMPs) is common^7^, ^13^, ^17^, ^35^, ^36^, ^37^ with some women perceiving CMPs to be safer to use in pregnancy and lactation than pharmaceutical medications.^28^, ^38^, ^39^, ^40^ Women also often take vitamin and mineral supplements due to the belief that supplementation will ensure they meet the additional nutritional requirements of pregnancy and lactation.^41^ In affluent economies, CAM use in pregnancy is greater in women with higher incomes, university education and is associated with primiparity.^24^, ^42^, ^43^ An important socio‐demographic component of health literacy is education, and advanced literacy and education levels have been shown to be strong predictors of positive health status.^44^, ^45^, ^46^ Whilst the demographic profile of most pregnant and breastfeeding CAM users in wealthy countries like Australia means they may not initially be considered to be at risk for limited functional health literacy,^47^ their actual health literacy levels have not been previously explored. In instances where self‐prescribing is common, functional health literacy may be particularly important.

Most pregnant or breastfeeding mothers want to promote their babies’ and their own health. Studies from Australia,^18^, ^28^, ^37^ and similar overseas economies^22^, ^48^, ^49^ confirm that safety of CMPs is very important to mothers. However, little is known about the role of health literacy in pregnant or breastfeeding women's decision‐making processes regarding the use of CMPs. A three‐tiered hierarchy of health literacy skills proposed by Nutbeam^50^ describes the skills consumers need to acquire, understand and use information when making health‐care decisions.^50^, ^51^ *Functional health literacy* skills are the first level and involve the reading, writing and numeracy skills required to understand factual health information regarding risks and medication prescriptions.^51^ The second level is *communicative health literacy* and requires more advanced cognitive and communication skills^50^, ^52^ to extract health information, apply it to different circumstances and communicate with health‐care practitioners (HCPs).^52^ Third, the most advanced level is *critical health literacy* whereby consumers’ skills are used to critically analyse and reflect information to support health‐care decisions.^50^, ^52^

This study aimed to explore the decision‐making processes pregnant and breastfeeding women go through when choosing to use CMPs from the perspective of the women themselves. It also aimed to investigate how women's health literacy skills influenced their decisions to use CMPs. Operational definitions used in this research appear in Box 1.

BOX 1 **Operational definitions**

1

- CMPs were defined as herbal medicines in ethanolic extract, tablet, capsule or tea form,^5^, ^53^ micronutrient supplements containing vitamins or minerals, and food supplements (eg probiotics or protein powders),^54^ topical preparations. CMPs could be purchased over the counter or after consultation with a HCP.^55^
- Women's health literacy needs were defined as the information needed and desired to make decisions about using CMPs in pregnancy and lactation, and the factors involved in obtaining and understanding this information.^56^

## 2 METHODS

### 2.1 Participants and recruitment

Purposive followed by snowball sampling approaches were used for recruitment and were directed at pregnant and breastfeeding women who used CMPs. This enabled the study aims to be investigated whilst ensuring that the sample was rich enough to enable participation from women of diverse experiences and backgrounds.^57^ The study was advertised on posters and flyers at playgroups, antenatal classes, pregnancy and postnatal yoga classes and support groups, in pharmacies and allied health practices, on free local classified advertising networks, and through [the Institution's] electronic media channels.

Women over the age of 18 who were currently pregnant and/or breastfeeding and who lived in the Northern Rivers region of New South Wales, or in the metropolitan regions of Sydney, Brisbane or the Gold Coast were invited to participate. Women also needed to be currently taking or have taken at least one CMP in the last 12 months and able to speak English well enough to participate in an in‐depth interview [IDI] or focus group discussion [FGD].

Women in the Northern Rivers area participated in face‐to‐face interviews and focus groups, FGDs and women at a distance from the interviewer participated in telephone or Skype interviews. All participants were given a $20 grocery voucher in recognition of their time.

Thematic saturation^57^ determined final sample size and was reached at 22 participants. An additional three interviews were held to confirm thematic saturation.

### 2.2 Study design

Qualitative methods were chosen to elicit in‐depth, detailed descriptions of the experiences, beliefs, values^58^, ^59^ and views of pregnant and breastfeeding women, and their motivators for using CMPs during pregnancy and breastfeeding. Qualitative methods allowed a compelling picture of the experience of CMP use to be collected and deepened understandings of these phenomena.^58^, ^59^ The use of both IDIs and FGDs allowed women to choose which format they would prefer and could participate in. This assisted with recruitment and enabled interviews to go ahead when FGDs were not achievable.

### 2.3 Data collection

A seven‐item semi‐structured interview guide was used during FGDs and IDIs (Table 1). Feedback from pre‐testing for face, content and construct validity from an interview with one pregnant woman, and a focus group with one pregnant and three breastfeeding women, was used to refine the questions. Pre‐testing also helped ensure that women who used CMPs in pregnancy and lactation had a voice in the design of the research.

**Table 1:** Guide for semi‐structured interviews and focus group discussions

Interviews and focus group discussion questions: Why do you use complementary medicine products? What sort of information do you want when considering taking complementary medicine products? What sort of information do you feel women who are pregnant or lactating need when considering using complementary medicine products? Where do you find the information you need when choosing to use complementary medicine products in pregnancy or whilst breastfeeding? What resources do you use? What do you feel would help pregnant and lactating women get the complementary medicines information they want and need to make safe decisions regarding using complementary medicine products? How easy is it for you to understand the information about complementary medicines you access? What would help you understand this information better? Can you please describe the decision‐making processes you use when choosing to take complementary medicine products?

All participants received an information sheet and had the opportunity to discuss the study before giving consent to participate. Participation was voluntary, and women could choose to withdraw from the study at any time. The decision to participate in an IDI or FGD was primarily the choice of the participant and depended on how comfortable the participant was in a group or individual setting, whether she wanted to bring her child/children to the IDI or FGD (babies and children were welcome), her work and family commitments, and distance from the interviewer‐researcher. The first author conducted all IDIs and FGDs.

Demographic details and data on women's use of CMPs at the time of the interview and in the previous 12 months were also collected.

Women's functional health literacy levels were measured using two validated health literacy screening tools. The first was the standard single question health literacy measure *How confident are you filling out medical forms by yourself?* 60 with response options: “extremely”, “quite a bit”, “somewhat”, “a little bit” and “not at all”. Those that chose “somewhat”, “a little bit” or “not at all” were considered to be at risk of inadequate health literacy.^60^, ^61^

The second was the *Newest Vital Sign*, a three‐minute direct test of consumer abilities that identifies people at risk of limited functional health literacy by measuring reading ability and interpretation skills, as well as aspects of numeracy necessary to understand nutritional information on food labels.^62^, ^63^ Participants who answered four or more of the six questions correctly were considered to have adequate functional health literacy, whilst a score less than two indicated that the participant had a large (>50%) chance of having inadequate health literacy skills.^62^, ^64^

### 2.4 Data analysis

The results from the demographic survey and health literacy assessment tools were analysed using descriptive statistics. All IDIs and FGDs were audio‐recorded and transcribed by an independent transcription service, then checked for accuracy against the original recording by LAJB. Transcripts were thematically analysed using the six steps of thematic analysis as described by Braun, Clarke^65^ using NVivo10 for data management. All transcripts were read multiple times to ensure a thorough understanding of the themes as they emerged, with themes grouped into major and minor subthemes. Constant comparison of findings was an essential part of the inductive thematic analysis, as potential codes and themes were identified, reviewed, defined, named, and refined, and relationships between themes identified. Participants shared information freely in IDIs and FGDs, including potentially sensitive data like complex health histories. The flexibility of the semi‐structured interview guide also facilitated the use of follow‐up questions within IDIs and FGDs, and in subsequent FGDs or IDIs to confirm the significance of the information. As no notable differences appeared between data from FGDs and IDIs, and between pregnant versus breastfeeding women, the data from all participants were grouped together for analysis. To increase validity, PA coded several transcripts and LAJB and PA met several times to review, discuss and agree on identified themes and subthemes for the final analysis. LB and KM also participated in reviewing and discussing the thematic analysis in final stages of the writing process. All participants were de‐identified and assigned pseudonyms for data reporting.

Additionally, LAJB kept a detailed research journal where ideas and themes from each interview and focus group were documented in an on‐going iterative process.

## 3 RESULTS

Between March and October 2016, a total of 25 women (n = 7 pregnant, n = 17 breastfeeding, n = 1 both pregnant and breastfeeding) participated. Three focus groups were held, one with two women, one with three women and one with four women. Nine women participated in individual face‐to‐face interviews, and seven women participated in Skype interviews. IDIs lasted for 40‐60 minutes; FGDs 70‐90 minutes.

### 3.1 Demographic information

Participants ranged in age from 23 to 40 years, and the average age was 32 years. Around half were first‐time mothers. Fourteen had between one and four older children, ranging in age from 2 to 11 years old. All women with older children reported having breastfed these children for 6‐34 months (mean 18 months). All but one woman completed the two health literacy screening tests (Table 2). This woman was unable to complete this section of the interview due to her baby waking. Most participants had good levels of functional health literacy according to the single item and NVS measures.

**Table 2:** Demographic profile of the participants

Demographic characteristics	Frequency (n)
Education	
Year 10 or equivalent	1
Year 12 or equivalent	2
Certificate 1‐4 level	4
Diploma	2
Bachelor's degree	8
Post‐graduate studies	8
Current employment status	
Full‐time home duties	3
On maternity leave from paid work	11
Part‐time employment	7
Full‐time employment	4
Income	
Low household income (AUD $475‐793 per week)	4
Medium household income (AUD $793‐1814 per week)	9
Higher income (> AUD $1815 per week)	11
Prefer not to answer	1
Relationship status	
Married or de facto relationship	24
Single	1
Birthplace	
Australia	14
New Zealand	5
United Kingdom	3
South Africa, the Netherlands, Colombia	1 each
Cultural and linguistic diversity	
Women who identified as being from non‐English speaking backgrounds	4
Women who identified as being from English speaking backgrounds	21
Smoking status	
Non‐smokers	25
Currently smoke	0
Health literacy levels	
Single item health literacy evaluation question: *how confident are you in filling out medical forms by yourself?*	
Extremely (not at risk** of** limited or marginal health literacy)	16
Quite a bit (not at risk **of** limited or marginal health literacy)	6
Somewhat (may be at risk of limited or marginal health literacy)	2
A little bit (inadequate health literacy)	0
Not at all (inadequate health literacy)	0
Newest Vital Sign	
Adequate functional health literacy (score 6/6)	18
Adequate functional health literacy (score 5/6)	3
Adequate functional health literacy (score 4/6)	2
Limited functional health literacy (score 3/6)	1

### 3.2 Complementary medicine use

Women listed the types of complementary medicines they currently took and had previously taken during their most recent or current pregnancy or breastfeeding journey (Appendix 1). A range of CMPs was reported. Pregnancy and breastfeeding multivitamin formulas were the most popular dietary supplements taken regularly across the sample. Probiotics, essential fatty acid supplements and iron supplements were also used widely. Consumption of herbal medicines during pregnancy was reported far less frequently than in lactation. The use of CMPs for breastfeeding issues and support was evident. Breastfeeding women reported using herbal teas and extracts to support breastmilk production and treat mastitis, and dietary supplements like lecithin to treat and prevent blocked milk ducts. A few participants reported using CMPs specifically chosen by their HCPs according to their specific health conditions.

### 3.3 Information sought in the decision‐making process

**Figure 1:**
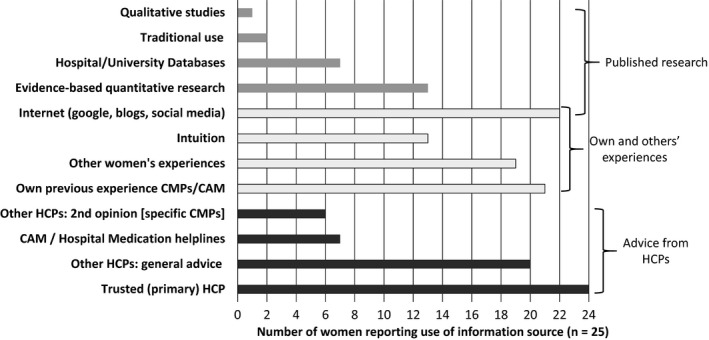
Three main areas where women sought information on complementary medicine products by frequency of use. CAM, complementary and alternative medicine and is inclusive of different CAM modalities; CMPs, complementary medicine products; HCP, health‐care practitioner

Women sought information from three main areas when deciding whether to take a CMP: HCPs, their own and other's experiences, and published research (Figure 1). Primarily, they wanted information from their most trusted HCP – usually midwives, naturopaths and integrative GPs, (medical doctors who combine conventional biomedicine and evidence‐based CAM in practice^66^) – but for some CMPs, they sought second opinions from other HCPs (pharmacists, naturopaths in pharmacies, health food stores or herbal dispensaries, and HCPs staffing CAM or hospital medication helplines). Although a few women mentioned having obstetric care, they did not identify their obstetricians as primary sources of CMPs information.

**Figure 2:**
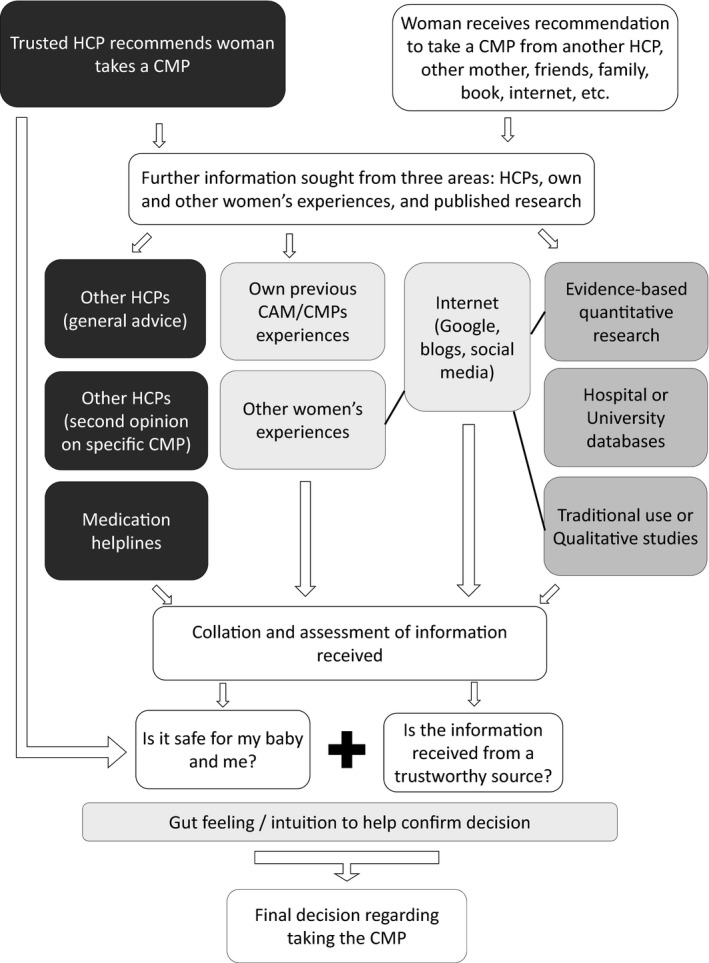
Women's decision‐making processes to use complementary medicine products in pregnancy and lactation. CAM, complementary and alternative medicine and is inclusive of different CAM modalities; CMPs, complementary medicine products; HCP, health‐care practitioner

Upon receiving a recommendation to use a specific CMP from their most trusted HCP, and being assured of its safety, some women immediately decided to take the CMP. However, if the recommendation was general (eg to take a pregnancy multivitamin), women searched for more information. This could include asking other HCPs for second opinions, discussing CMPs with other women, and looking for published research and other written information on the Internet and in books. Often the search for more information involved comparing similar CMPs to find what they felt was the best quality product, always keeping in mind the safety considerations. Women also reported obtaining information about, or a recommendation to use a CMP, from other sources, including family, friends, their own reading or background knowledge, and would use the same strategies to search for more information. Intuition was an element of decision making mentioned by around half the participants, most often for CMPs not specifically recommended by their trusted HCP. These women reported that they used their ‘gut feelings’ in the final stages of decision making after collating information from multiple sources, checking what was known about a CMPs safety and assessing the trustworthiness of their information sources (Figure 2).

If it's recommended by the GP or nutritionist with the reasons why I might need to take it …And then I get some advice from the chemist or the pharmacist. I’ll ask friends and colleagues, other pregnant and breastfeeding women what they’ve done, if they've heard about it, if it's helped them. I’ll try and get good information. Obviously, I make an informed decision and then go on that gut feeling whether it’s going to work for me or not. (Halley, breastfeeding mother)

**Figure 3:**
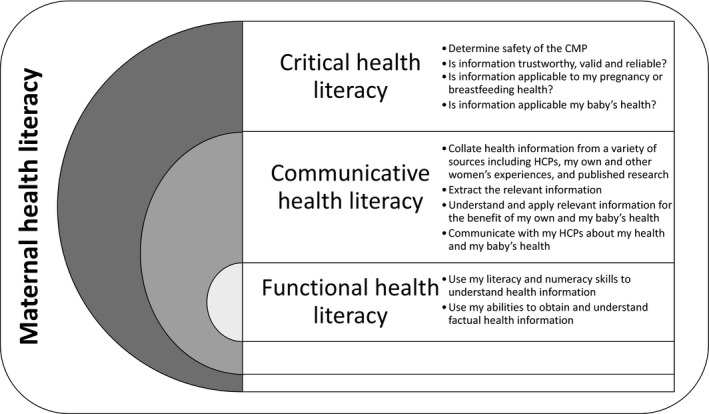
Functional, communicative and critical health literacy as demonstrated by participants within the overarching concept of maternal health literacy, defined as ‘the cognitive and social skills that determine the motivation and ability of women to gain access to, understand and use information in ways that promote and maintain their health and that of their children.^56^ ^,p381^ CMP, complementary medicine product; HCP, health‐care practitioner

Figure 3 describes the skills used, actions taken and questions women asked during their decision‐making processes and analysis of information. These are grouped into the hierarchy of health literacy classifications outlined by Nutbeam,^50^ within the overarching concept of maternal health literacy.^56^

### 3.4 Factors influencing the decision‐making process

Thematic analysis determined two overarching components of the decision‐making processes (Table 3): (1) Women's information needs and (2) Preference for CMP use. Regarding *Women's information needs*, two major themes with associated subthemes were identified: (1a) Ensuring safety for the baby and the mother and (1b) Seeking information from a trusted source. Regarding how *Preference for CMP use* influenced the decision‐making processes, three themes were identified: (2a) Supporting pregnancy and breastfeeding health for mother and baby; (2b) Past experience with CMP use; and (2c) Desire for holistic health care. The overarching themes integrate, influence and determine a woman's decision making to use CMPs during pregnancy and/or breastfeeding.

**Table 3:** Women's information needs and underlying influences regarding the decision‐making process to use CMPs in pregnancy and lactation: major themes and subthemes

Themes	Subthemes

1a) Ensuring safety for the baby and the mother	Assessing possible harms to the baby Assessing possible harms to the mother Understanding the actions of the CMP in my body Dosage and timing considerations Assessing possible benefits for the mother Collating information from multiple sources to assess safety
1b) Seeking information from a trusted source	Receiving information on CMPs from trusted health‐care practitioners The importance of the therapeutic relationship in engendering trust Assessing information from other sources o Information from other health‐care practitioners o Other women's experiences o Research
Overarching component 2. Preference for CMP use	
2a) Supporting pregnancy and breastfeeding health for mother and baby	Optimizing my own health o for mother's benefit o to support baby's health in utero or through breastmilk Optimizing my baby's health Optimizing both the mother's and the baby's health Using CMPs for specific health conditions Use of CMPs to support well‐being during breastfeeding, for milk supply, and cracked nipple pain
2b) Past experience with CMP use	Long‐term use of CAM/ CMPs Complex health histories
2c) Desire for holistic health care	Complementary medicine as a preferred first course of treatment o Distrust of pharmaceutical medications o CMPs are natural and therefore safe

### 3.5 Overarching component 1. Women's information needs

#### 3.5.1 Ensuring safety of the baby and the mother

Safety was of prime importance to all participants. Most demonstrated a critical approach in using five key questions to assess the safety of a CMP. They wanted to know (a) whether the CMP could harm their baby, (b) be of benefit to their baby, (c) be of benefit to themselves, (d) support a healthy pregnancy or breastmilk production and (e) what actions the CMP had in the body.

All participants expressed the desire to know whether a CMP would harm their unborn or breastfeeding baby and most (21/25) wanted to know that the CMP was also safe for them. Women often double‐checked safety with their trusted HCP, even if the HCP had recommended the CMP, and sometimes used multiple sources (eg Internet, asking HCPs when purchasing over‐the‐counter products, medication helplines) to further assure themselves of safety.

I never take anything without either double checking, online and asking a professional about it as well, to make sure that what I’m planning to do isn’t going to have detrimental effects, a) when I was pregnant and b) whenever I'm breastfeeding. (Cara, breastfeeding mother)

When discussing their CMPs, it was evident that women treated their CMPs as medicines, as they often spoke about safety concerns with pharmaceutical medications at the same time as discussing CMPs. Some mothers also expressed that they avoided taking some CMPs or pharmaceutical medications during pregnancy and breastfeeding, for fear it would affect the baby, themselves or their milk supply.

I need to know that [CMPs] have no adverse effects on me or the milk supply or that it goes through my milk to the baby… the same with taking anything throughout the pregnancy. I did have morning sickness but I wouldn't take anything for it. I'm not going to risk it. (Halley, breastfeeding mother)

Some women also recognized that scientific evidence was not always available for the CMPs they chose to take and relied on finding information about traditional use from HCPs, other women's experiences and published studies to confirm safety.

A girlfriend of mine told me about raspberry leaf and nettle tea, so I spoke to the naturopath in the health food shop, and other women, and I read more about them in a natural birth book. I didn’t find any scientific proof that they worked. (Joni, breastfeeding mother)

If there’s any evidence‐based information I try to find it. But I know with complementary medicine, often there's not enough research for it to be evidence‐based. So, a lot of it can be anecdotal or qualitative studies or traditional use. (Marley, breastfeeding mother)

Dosage was an important aspect of safety considerations. Women were concerned that they not overdose on any herbs or micronutrients, and wanted to know appropriate dosage regimes.

The desire for knowledge around the actions of a CMP was a strong theme that was tied to women's safety considerations. Most women (22/25) wanted to know what actions the CMP had in the body, possible side‐effects, and whether a CMP could be used for more than one condition.

Effectiveness was also an important consideration for women and they collated information from multiple sources to assess this:

Whether it's the GP, the obstetrician, my nutritionist, the chemist, the pharmacist who tell me about [a CMP], I then ask friends and colleagues or other pregnant or breastfeeding women what they've done. And then, I might Google and see what information is there. (Halley, breastfeeding mother)

Women also expressed finding information about the actions of a CMP useful when considering whether or not it would interact with other CMPs and how best to schedule taking those CMPs that impacted on absorption of other CMPs.

Something that was really helpful having a naturopath for, was knowing what interacts with what. She helped me organise my schedule of when to take what. I take calcium in the mornings and then my iron two hours later. She told me not have iron and calcium together because calcium can inhibit iron absorption. (Kim, breastfeeding mother)

### 3.6 Seeking information from a trusted source

Women wanted to receive information about CMPs from sources they trusted. All but one participant sought information from her trusted HCP, who were predominantly CAM practitioners, midwives or integrative doctors, demonstrating knowledge of complementary medicine.

I’m lucky to have a very holistic GP. She endorses all sorts of complementary medicines, and says, ‘These ones are what you should think about. Maybe don’t try that.’ Her advice is always lifestyle, exercise, improving diet. It’s nice to have someone that wants you to eat right before you start popping pills. So, I trust her advice as well. (Joni, breastfeeding mother)

The therapeutic relationship with the trusted HCP was very important for participants and contributed to the trust they felt in their naturopaths, midwives or integrative GPs.

I’ve trusted in what she’s [naturopath] said. I’ve been with her for six years and she’s done incredible stuff with my health. She’s got years of expertise and I make sure she knows when I’ve been pregnant or breastfeeding. So, I take what she prescribes me. (Vanessa, breastfeeding mother)

Within the therapeutic relationship, women felt they could be active participants in their own health, ask questions about suggested treatments, be heard and have their opinions and preferences valued.

Participants rated the information received from lay sources, including other pregnant or breastfeeding women, family and friends, and information read in books or online, lower than information received from their trusted HCPs. Women searched for more information about CMPs if they felt the recommendation was important enough to consider finding supporting evidence.

So, someone will recommend [a CMP] and I’ll still take into account my own feelings towards it… I don't really trust blogs and websites either. I take advice with a grain of salt, and then do my own research. (Donna, pregnant mother)

Women used four key questions to determine trust in information obtained from HCPs and lay sources: qualifications of the person offering the advice; their experiences with CMPs; the evidence they had for recommending a CMP; and their motivation for recommending a CMP. Encountering the same information or advice from multiple sources enabled women to check the evidence base for initial recommendations received from lay sources and increased their confidence in their final decision making. Marley displayed a high level of critical literacy and captured this well:

I look at websites and check if it’s endorsed by anyone in particular. Who is it written by? A naturopath, a doctor, other health professional? Or is it written by someone that’s just dabbling in naturopathy? I look at their credentials… I ask, ‘Why are they promoting that? Is it because of personal experience or is it because they're wanting to make money out of it? Or is it because it's worked for them, but they haven't actually tried it on anyone else?’ And then reading the article and just going through the research. Does it make sense? Is it logical? Unbiased? Is it saying both the positives and benefits and negatives of the herb or medicine? (Marley, breastfeeding mother)

### 3.7 Overarching component 2. Preference for CMP use

Most women expressed a preference for complementary medicines, viewing use of CMPs as a normal part of health care, although they also sought maternity care from biomedically trained midwives and doctors. Previous significant experience of and preference for CAM or CMPs were not a factor in decision making for only three participants. The remaining 22 participants’ decisions to use CMPs were connected to their aims to optimize health in order to have a healthy pregnancy and a healthy baby. Women's past experiences with CMP use and desires for holistic health care were evident themes influencing women's decision making to use CMPs in pregnancy and lactation.

### 3.8 Supporting pregnancy and breastfeeding health for mother and baby

Women took specific CMPs to support their babies’ health during pregnancy (15/25) and whilst breastfeeding (8/25). Women also reported taking CMPs to help optimize their own health and thus their baby's health. In pregnancy, this usually involved taking folic acid and/or pregnancy multivitamins, but some women used specific CMPs for their individual health conditions. During breastfeeding, some women took CMPs with the aim of providing prophylactic immune support for their breastfeeding children through their milk. A few women were investigating or using CMPs as galactagogues, due to diagnosed or perceived milk supply issues. They also commonly spoke about using CMPs to treat breastfeeding‐specific medical problems like cracked nipples, blocked ducts and mastitis, and appreciated that the use of CMPs helped them to continue to breastfeed successfully.

One duct would keep getting blocked, so I got a herbal tincture from the herbalist, and pokeroot cream to loosen it, break it up, to help move it, so I could keep breastfeeding. (Bella, breastfeeding mother)

### 3.9 Past experience with CMP use and desire for holistic health care.

For half the sample, women's preference for CMP use was also related to having used complementary medicines for the majority of their lives (12 participants), and for three participants, to help with fertility challenges. Many (17/25) reported quite complex health histories and the use of complementary medicine to resolve or positively improve health issues. Their trusted HCPs had helped them through their health journeys, and the improvements in health experienced contributed to the trust they felt in their HCPs.

I originally started taking [CMPs] because I was diagnosed with an underactive thyroid and became quite unwell, rapid weight gain, a lot of fatigue, all my hair falling out, carpal tunnel. A lot of full‐on symptoms really quite quickly. So, I found a naturopath because mainstream medication wasn’t working and I saw a massive improvement in only a couple of weeks! So, I’ve continued seeing [naturopath] and using [CMPs] during pregnancy to make sure my thyroid remains stable, but also for growing a healthy baby, and now, to get the right vitamins and minerals for breastfeeding and to maintain my health as a new mum. (Vanessa, breastfeeding mother)

Finally, women's desires for holistic health care also contributed to their decision making to use CMPs, with CMPs being a preferred first course of treatment for many, and expressly culturally normal for some:

You have your family and everybody telling you, ‘You can use this. You can use that’. In Colombia, we use herbs from the backyard. It's normal for us because it's traditional medicine, it's your inheritance from the families… There are many natural things that you can use and they are better for you and [it] is less process[ed]. I think if I'm pregnant or if I have a baby, I would be happy to have something natural… (Gabriela, pregnant mother)

A few also expressed a distrust of pharmaceutical medications, usually because of safety reasons, contrasting this with the perception of CMPs as natural and therefore safe.

## 4 DISCUSSION

Most participants in this study had high levels of functional health literacy, as shown by the Newest Vital Sign^62^ results and single item health literacy measure.^60^, ^61^ The demographic profile of the participants, especially their high education and income levels, also reflects what has been previously shown about typical Australian women who use CMPs in pregnancy and lactation.^14^, ^18^, ^35^, ^36^ Most participants demonstrated sophisticated analytic skills during their decision‐making processes and showed high communicative and critical health literacy skills in the questions they posed and sought to answer. These factors led to women engaging in very complex decision‐making processes when choosing to use CMPs during pregnancy or breastfeeding. This decision‐making process involved seeking, collating and assessing information from HCPs, their own and other women's experiences, and published research (Figure 1), before making an informed decision, based on perceptions of safety and trustworthiness of information (Figure 2). In line with the concept of maternal health literacy,^56^ women's decision‐making processes reflected a need to make health‐enhancing decisions for themselves and their children. There were no notable differences between the decision making in pregnancy or breastfeeding, primarily because the motivations behind the decision‐making process were similar, especially the need to establish the safety of a CMP in order to ensure the health and well‐being of their unborn or breastfeeding children.

### 4.1 Women's communicative health literacy

Communicative health literacy describes a person's motivations, confidence and abilities to act independently on health knowledge,^50^ interpret health information meaningfully and apply it in different circumstances.^51^ Participants in this study demonstrated high communicative health literacy in several ways (Figure 3), including collating CMP‐related information from multiple sources. Previous research has identified many similar information sources to those used by participants in this study, and the use of plural resources by mothers when seeking information about CMPs.^14^, ^18^, ^25^, ^67^, ^68^, ^69^, ^70^ However, this study identified that women did not rate the information received from family, friends, peers and the Internet as highly as that received from trusted HCPs who had qualifications and experience in CAM modalities. Whenever possible, women preferred to determine a CMPs safety and indications through discussions with their trusted HCPs. Women used multiple sources of information to determine the quality of information obtained. Shared social bonds may be an important influence on self‐prescription^9^, ^71^ and are evident in other studies on CMP use where pregnant or breastfeeding women share CMPs information with each other and receive CMPs information from their family, friends and HCPs.^13^, ^17^, ^18^, ^28^, ^29^, ^36^, ^37^, ^49^, ^72^ In this study, the sharing of information both in person and in online forums with peers was an important consideration in the decision‐making process,^29^ especially when participants described receiving recommendations for CMPs from several non‐HCP sources.

The second major demonstration of communicative health literacy in this study was participants’ active engagement in discussions with their HCPs to obtain CMPs information and safety profiles. Shared value systems with their HCPs and longer consultation times, both of which have been noted as core components of complementary medicine and integrative care,^32^, ^73^ facilitated the discussion of different treatment options and associated potential consequences and were highly valued by the participants. The relationship between a pregnant or breastfeeding woman and her HCP is a very important part of maternity care^74^, ^75^; however, the emphasis women in this study placed on the importance of receiving CMPs information from trusted HCPs may not have been fully explored before, especially as it pertains to the therapeutic relationship and women's preferences for holistic health care.

Participants’ trusted HCPs’ embracement of holistic practice also aligned with their desires for positive therapeutic relationships with their HCPs. Women highly valued their HCPs’ holistic consideration of their own and their baby's health, and having all their experiences and values considered. This holistic approach has previously been identified as an important element of care provided by complementary and integrative medicine practitioners,^76^, ^77^ including their care of pregnant women,^31^ as well as woman‐centred midwifery practice.^74^, ^78^ When an accomplished HCP is able to understand a woman's experiences and beliefs and take these into consideration when constructing a plan to optimize her health, a positive therapeutic relationship is supported.^79^ Considering that most Australian women seek biomedical care during pregnancy^80^ and that high use of complementary medicines in pregnancy has been noted,^20^, ^35^ it is not surprising that for this study, like others, biomedical HCPs were identified as important sources of CMPs information in pregnancy,^43^ especially when these HCPs were integrative practitioners and demonstrated some knowledge and experience with CAM. However, this finding does contrast with other research that shows it is uncommon for women to engage in discussions regarding their CMP use with their biomedical practitioners, either because these practitioners do not ask about CMPs,^24^, ^81^ women do not consider discussing CMPs with them,^82^ or they fear negative reactions from their doctors or midwives if they raise CMP use with them.^22^, ^47^, ^48^, ^83^ The positive therapeutic relationship identified between study participants and their trusted HCPs was a key factor in women's perceptions of the high quality of information received from these HCPs. This is especially important to note when considering women's primary desire to know that the CMPs they chose to take were safe.

### 4.2 Women's critical health literacy

Critical health literacy builds on communicative health literacy and describes how well an individual can analyse and consider health information and use it to increase their autonomy in health‐care choices and other life events.^50^, ^52^ The ways women evaluated the CMPs information they collated to determine whether it was trustworthy, valid and reliable was a key component of the way they demonstrated their critical health literacy skills (Figure 3). Determining a CMPs safety in pregnancy or breastfeeding was imperative and frequently drove participants’ complex information‐gathering processes, especially if they received CMPs information from a source other than their trusted HCPs. In order to validate their trusted HCPs CMP recommendation, many participants gathered information from multiple sources and sought information from at least three sources before making their final decisions (Figures 1 and 2). This high level of critical health literacy reflects the women's keen engagement with their own health and the ‘active consumer’ noted in CAM users previously.^43^

Using critical health literacy skills to evaluate CMPs information also required women to assess whether the use of the CMP was applicable to their own or their babies’ health and required some complex assessments due to the limitations in empirical evidence available for some CMPs. Participants were willing to acknowledge the validity of evidence for safety and/or efficacy of CMPs outside the limits of evidence‐based testing,^84^ especially if a CMP was endorsed by their trusted HCPs who were seen to have knowledge and expertise in CAM. Women identified that a CMPs safety profile, especially for herbal medicine, may only be established through knowledge passed down through centuries and corroborated by their trusted CAM or integrative HCPs.^84^ This reliance on traditional knowledge for evidence of safety and efficacy is necessary considering the small numbers of published clinical trials that look at herbal medicine use in pregnancy^85^ and lactation.^86^

### 4.3 Strengths and limitations

Using two validated assessments of health literacy levels demonstrated greater reliability of results regarding participants’ health literacy levels. Nevertheless, an important limitation was that all but one participant in the sample demonstrated high functional health literacy skills, and the entire sample showed sophisticated communicative and critical health literacy skills, which may explain their extensive information seeking and complex decision‐making processes. This study does not represent the full range of health literacy levels and further research on CMP use with lower health literacy samples is needed, especially considering that qualitative research cannot be generalized outside the study sample. However, this limitation can also be considered a strength of the study, as the demographics of the study sample reflect the typical Australian woman who uses CMPs in pregnancy or breastfeeding.^14^, ^18^, ^35^, ^36^ Investigating CMP use in a sample of pregnant of breastfeeding women with high health literacy and education levels has enabled deep insights into the decision‐making processes of these women who use CMPs in pregnancy and lactation. It may be difficult to find Australian pregnant or breastfeeding women with lower health literacy levels who use CMPs. Additionally, whilst the hierarchy of functional, communicative and critical health literacy levels^50^ has been examined in populations living with diabetes and other chronic disease,^51^, ^52^ future research is needed to advance knowledge in the area of health literacy and maternal decision making regarding CMP use in pregnancy and lactation. Social desirability may also have influenced some participants’ responses, if they were unwilling to report use of CMPs without any decision‐making processes. Another possible limitation in the sample relates to the participants’ interest in CAM and motivation to participate, which could have contributed to a sample with more information seeking styles.

## 5 CONCLUSIONS

Women's decision‐making processes were quite complex and involved assessments of safety and quality of information, and reflected their high levels of health literacy. Participants were aware that taking CMPs could positively or negatively affect the health of their babies and themselves, and sought to manage this risk by seeking information on the safety of CMPs. They considered various levels of evidence regarding CMPs’ safety and efficacy, preferring to receive such information from trusted HCPs with whom they enjoyed and valued positive therapeutic relationships. Another important influencing factor on the participants’ decisions was their positive attitude towards CAM and a health‐care outlook that embraced supporting the optimization of health and well‐being. Fostering good therapeutic relationships between HCPs and women during maternity care creates an opportunity for open discussion and a critically informed approach to CMP use in pregnancy and lactation, which ultimately may enhance woman‐centred maternity care.

## CONFLICT OF INTEREST

Philanthropical funding from Blackmores Ltd. funded Larisa Barnes’ PhD scholarship during the course of this research. However, Blackmores has had no input into the design, execution or the dissemination of her research. The authors declare that they have no conflicts of interests.

## ETHICAL APPROVAL

This study received approval from the University of Sydney's Human Research Ethics Committee (approval number 2015/730).

## DATA AVAILABILITY

The data sets generated and analysed during the current study are not publicly available as participants did not consent to transcripts of interviews and focus group discussions being shared. Additional details relating to other aspects of the data are available on reasonable request from the authors.

## APPENDIX 1

### CMPs women reported using during pregnancy and lactation

Participants reported using a range of CMPs during pregnancy and breastfeeding, including ingested CMPs and topically applied CMPs.

Pregnant women reported on their current use of CMPs, and the CMPs they had previously used in their current pregnancies.

Breastfeeding women reported on their use of CMPs in their most recent pregnancies, as well as their current and previous use of CMPs during their current breastfeeding journey.

**Table:**

	CMP use during pregnancy			CMP use during breastfeeding	
	Number of pregnant women currently taking CMP	Number of pregnant women reported having taken CMP previously during present pregnancy, but not currently	Number of currently breastfeeding women who reported taking CMP during their last pregnancy	Number of breastfeeding women currently taking CMP	Number of currently breastfeeding women who reported taking CMP at some point during present breastfeeding experience, but not currently
Vitamin and mineral supplements (ingested)					
Pregnancy and breastfeeding multivitamin	7	6	15	12	0
Essential fatty acid omega 3 supplements (fish oils or vegetarian)	5	5	8	6	0
Probiotics	3	4	11	10	2
Iron	3	2	12	4	0
Combination of iron and folic acid supplement	0	0	0	1	1
Iodine	1	1	2	0	0
Folate or folic acid	2	3	9	0	0
Vitamin D	1	1	4	1	0
Other supplements^a^	5	8	21	21	21
Herbal medicines (ingested)					
Raspberry leaf tea or supplements	1	2	12	0	0
Ginger tea	1	1	0	0	2
Other herbal medicines^b^	3	2	8	10	18
Topically applied CMPs					
CMPs applied to skin (herbal compresses, creams, sprays and lotions, massage oils)	0	4	1	3	14
Essential oils used in oil burners for relaxation, nausea, or other purpose	0	3	1	2	3
Homoeopathic and tissue salts remedies					
Homoeopathic or tissue salts remedies^c^	0	0	2	4	2

a Other supplements included evening primrose oil, calcium, magnesium, vitamins C, B complex and B6, zinc, selenium, lecithin, brewer's yeast, a mixed vitamin‐mineral thyroid support tablet, glutathione, calcium di‐gluconate.

b Other herbal medicines included herbal extract blends and supplements individually prescribed by participants’ HCPs for various health conditions, several different herbal teas taken to support breastfeeding (supply and to treat/prevent mastitis), and/or to support digestion.

c Used to treat nausea, mastitis, for labour induction or unspecified reason.
